# A step-by-step guide for conducting an umbrella review

**DOI:** 10.1186/s41182-025-00764-y

**Published:** 2025-10-09

**Authors:** Mohammed Abdellatif, Mohammad Najm Dadam, Nguyen Tuan Vu, Nguyen Hai Nam, Nguyen Quoc Hoan, Zahraa Taoube, Phillip Tran, Nguyen Tien Huy

**Affiliations:** 1 Pediatric and Neonatology Specialist, Muscat Private Hospital, Muscat, Oman; 2 Online Research Club, Nagasaki, Japan; 3 Department of Orthopedics and Trauma Surgery, Helios Klinikum Schwelm, Schwelm, Germany; 4 Ho Chi Minh City Eye Hospital, Ho Chi Minh, Vietnam; 5 Department of Liver Tumor, Cancer Center, Cho Ray Hospital, Ho Chi Minh, Vietnam; 6 School of Dentistry, Hanoi Medical University, Hanoi, 100000 Vietnam; 7 MSH Medical School Hamburg, Hamburg, Germany; 8 Invasive Cardiology, Nam Can Tho University, Can Tho, Vietnam; 9 Institute of Research and Development, Duy Tan University, Da Nang, Vietnam; 10 School of Medicine and Pharmacy, Duy Tan University, Da Nang, Vietnam; 11 School of Tropical Medicine and Global Health, Nagasaki University, Nagasaki, Japan

**Keywords:** Umbrella review, Evidence-based practice, Meta-reviews, Review of reviews, Evidence synthesis

## Abstract

**Background:**

The rapid increase in published research poses challenges for healthcare professionals aiming to stay updated. While systematic reviews help synthesize findings, their abundance can overwhelm readers. Umbrella reviews address this by summarizing multiple systematic reviews, offering a comprehensive perspective on specific topics.

**Methods:**

This article offers a practical, step-by-step guide for conducting an umbrella review, aimed at researchers and clinicians alike.

**Results:**

Umbrella reviews effectively integrate data from systematic reviews, ensuring clearer evidence synthesis. Tools like Rayyan and Covidence streamline processes such as screening and data extraction. Strategies for managing overlapping studies and assessing methodological quality enhance the validity of findings.

**Conclusion:**

Umbrella reviews are invaluable for evidence-based decision-making, especially in healthcare. This guide equips researchers and clinicians with a structured approach to navigate and synthesize the growing body of systematic reviews, fostering reliable and actionable insights.

**Supplementary Information:**

The online version contains supplementary material available at 10.1186/s41182-025-00764-y.

## Introduction

In modern medicine, evidence-based decision-making is crucial [1], driving the need for reliable information amid an exponential growth of research articles [2], which makes it difficult for practitioners and researchers to stay up-to-date [1]. In response to this challenge, systematic reviews emerged to consolidate vast scientific literature into concise summaries [2]. However, the rapid increment of systematic reviews itself posed a fresh set of challenges for readers [3], which underscores the need for a more comprehensive approach: the umbrella review.

In one sentence, an umbrella review can be seen as a review of systematic reviews. They may also be referred to as “overview of systematic reviews,” “meta-reviews,” or “review of reviews.” By collating and interpreting data addressing a few predefined questions, umbrella reviews prevent researchers from being overwhelmed with large volumes of contradictory individual evidence [4–6]. These reviews also assess the quality of evidence and potential publication bias in the included reviews, fostering evidence-based decision-making in clinical practice [7].

Current standard recommends following the Preferred Reporting Items for Overviews of Reviews (PRIOR) checklist, to ensure that the review process is rigorous, transparent, and reproducible. The PRIOR checklist covers all essential steps from the rationale to data synthesis (Supplementary Table 1) [8]. However, the PRIOR checklist does not explicitly present a step-by-step approach to conduct an umbrella review, which is a challenge to those who has no experience in conducting such reviews. This article provides a step-by-step process of conducting an umbrella review, highlighting its advantages and potential as a reliable source of evidence for healthcare decision-making, following the PRIOR guidelines. To further illustrate the steps of conducting an umbrella review, we used an article entitled: “Dietary Sugar Consumption and Health: Umbrella Review”[9].

## Steps for conducting any umbrella review

**Fig. 1:**
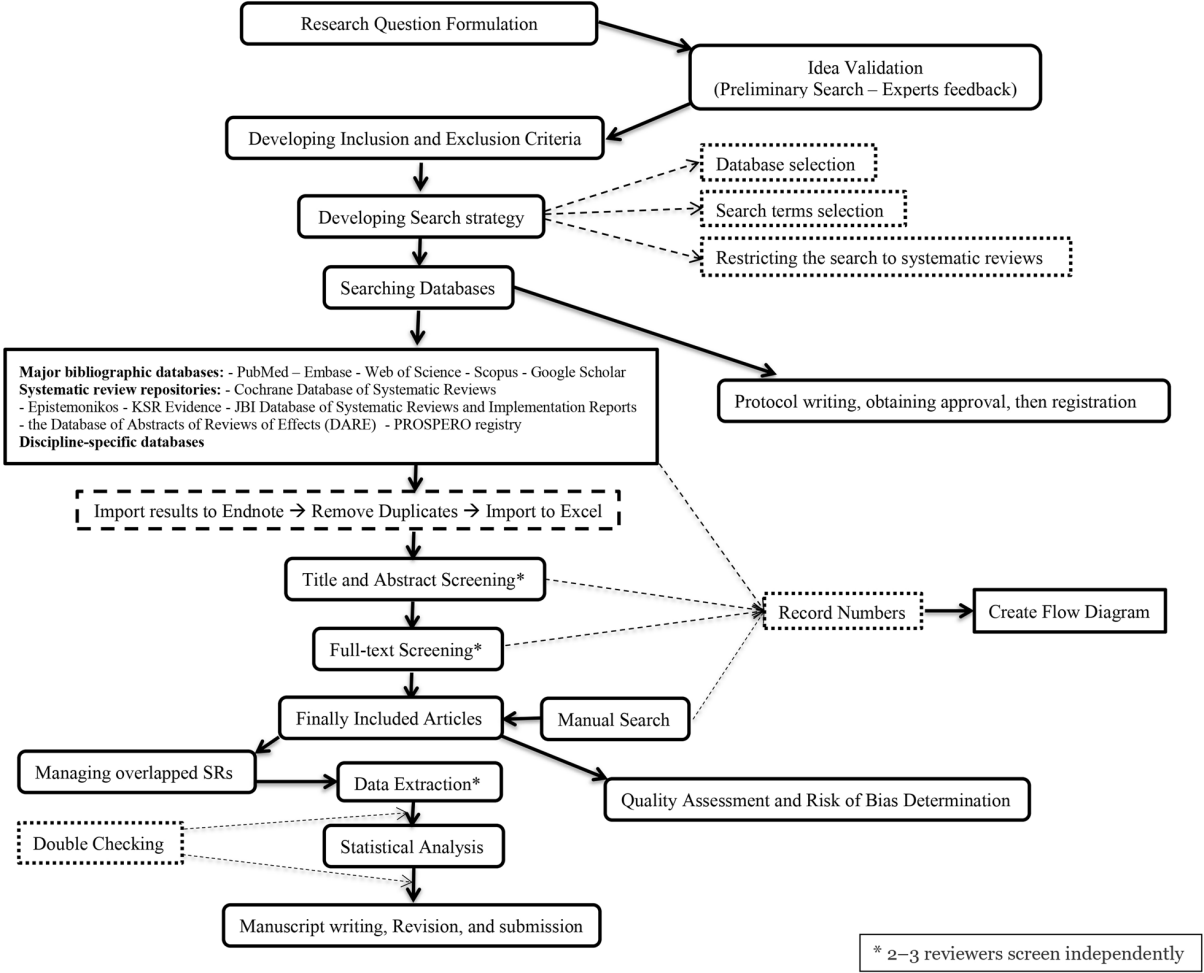
Detailed flow chart guideline for umbrella review steps

Figure 1 shows a detailed flow chart for umbrella review steps

### Formulating the research question

Formulating a research question is the cornerstone of building strong and reliable research. This involves the following steps:

Determine the broad topic or overarching theme, such as a medical condition, a population group, or an intervention.

To narrow down the question, systematic frameworks such as PICO (Population, Intervention, Comparison, and Outcome) are commonly used. However, the choice of framework should align with the review’s focus—for example:

- **PICOC** (Population, Intervention, Comparison, Outcome, *Context*)
- **CoCoPop** (Condition, Context, Population) for prevalence/etiology reviews.
- **PEO** (Population, Exposure, Outcome) for questions related to qualitative data.

Clarify the objectives, such as comparing interventions, evaluating effectiveness, or assessing safety profiles.

Determine the review’s scope, including time periods, regions, or study designs. Set inclusion/exclusion criteria.

Determine if specific aspects or subgroups, such as different dosages or administration methods, should be included.

Distill key elements into a concise question. Include the PICO framework and contextual factors.

### Ensure the feasibility and relevance of the idea

The authors should then ensure feasibility by assessing the availability of relevant systematic reviews. Then seek input and feedback from colleagues and experts to ensure relevance and quality.

### Developing inclusion and exclusion criteria

Inclusion and exclusion criteria should stem from the selected framework, which outlines the relevant characteristics of included studies [8, 10, 11]. In an umbrella review, only SRs with or without meta-analyses, are included [12]. It is crucial to define a systematic review clearly, as not all publications labeled as such meet the criteria, while some that aren't explicitly labeled may qualify [11]. Researchers need to decide whether to include only SRs of randomized controlled trials (RCTs) or to incorporate other designs, such as observational studies, depending on the research question and available evidence [11].

### Database search for systematic reviews

Database search is a crucial step in conducting umbrella reviews. It involves careful selection of databases, choosing appropriate search terms, and restricting the search to focus on systematic reviews.

#### Database selection: this includes

- Major bibliographic databases such as PubMed/MEDLINE, Embase, Scopus, and Web of Science [13]
- Systematic review repositories such as the Cochrane Database of Systematic Reviews, Epistemonikos, KSR Evidence, JBI Database of Systematic Reviews and Implementation Reports, DARE and the PROSPERO register [11, 12]
- Discipline-specific databases like PsycINFO, LILACS or CINAHL [11]

#### Selecting search terms

Researchers need to carefully choose relevant search terms, including controlled vocabulary terms (e.g., MeSH) and free-text terms [10, 11].

The search terms should accurately reflect the research question formulated earlier using the PICO framework and the concepts being studied.

#### Restricting the search to systematic reviews

The aim is to optimize the search strategy by minimizing the inclusion of non-systematic review publications [14–17]. This can be achieved by:

- Using specific search filters or limiters, such as the "Systematic Reviews" and “Meta-Analysis” filters in PubMed.
- Utilizing MeSH headings and search terms specifically targeting systematic reviews, such as "systematic review", "review literature", "literature review", "systematic* review*", "systematic* literature*", "systematic* search*", "systematic* synth*", "systematic* identif*", “systematic overview", "meta-analysis", and “metaanalysis”.
- In addition, databases such as the Cochrane Database of Systematic Reviews should be included to ensure comprehensive coverage of high-quality SRs.

Deciding which SRs to incorporate into the UR holds significant influence over the review process. To ensure accuracy, at least two authors should work independently to assess whether each study aligns with the eligibility criteria [18–20].

### Selecting eligible systematic reviews

#### Selecting eligible SRs involves the following steps

- Combining search results from different databases into one list
- Screening titles and abstracts
- Downloading the full text of the potentially relevant reports.
- Screening the full texts against eligibility criteria
- Resolving possible conflicts through discussion
- Reach a consensus on study inclusion and record the whole process in the flow diagram

#### Remember

- Failure of one inclusion criterion leads to exclusion of the review
- If a potentially relevant SR has a broader research question than the UR, evaluate the primary studies included in this review against the UR’s inclusion criteria

**Fig. 2:**
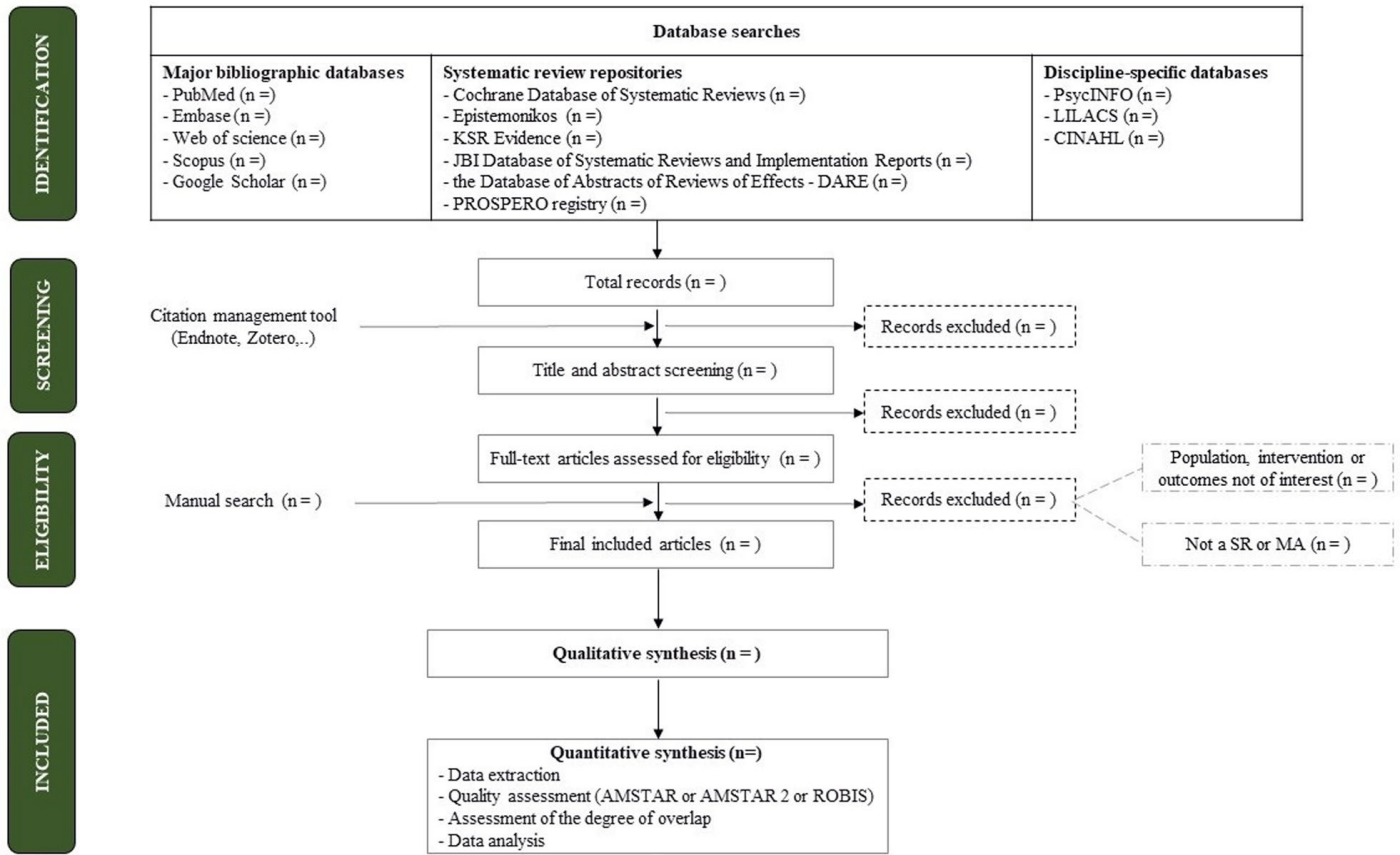
Flow Diagram of Systematic Reviews Identification and Selection for the Umbrella Review

The entire database search process and systematic reviews selection should be thoroughly documented with precise details. Figure 2 shows a proposed flow diagram of systematic reviews identification and selection for the umbrella review.

### Data collection, analysis, and presentation from included systematic reviews

Once the relevant systematic reviews have been identified, data collection, analysis, and presentation can be carried out.

#### Data collection

Initially, a pilot extraction sheet should be generated using random reviews. If necessary, subsequent modifications should be discussed among all reviewers [11, 12].

Two to three researchers should independently extract the required data using a standardized data extraction form. In the event of any disagreements, they should be resolved through discussion and consensus [10, 11].

Data extraction should encompass information regarding SRs and the primary studies they comprise. Generally, these data can be categorized into four main groups: basic characteristics, quantitative outcomes, data for evaluating the risk of bias, and data for assessing the certainty of evidence [17, 21]. Box 1 contains data that are typically extracted from the included articles.

### Box 1: Data typically included in the data extraction form

- Authors’ names
- Publication year
- The number of databases searched
- Search time period
- Review objectives (primary and secondary outcomes)
- Interventions and control arms (type, dose, intensity, frequency, and duration)
- Results synthesis method
- Key findings
- Types of studies contributing to outcomes and their countries of origin
- Quality assessment instrument and ratings of each systematic review as well as the included primary studies.
- Basic information about the included primary studies

Authors should plan ahead for missing or inadequately reported data [17, 21]. They can either acknowledge data gaps in their umbrella review or extract missing data from primary studies. Extracting a significant amount of data from primary studies may prompt authors to consider a systematic review instead of an umbrella review [11].

For systematic reviews without meta-analyses, the rationale for not conducting MA should be stated [10].

#### Data analysis and presentation

There are two main ways to present outcome data in an umbrella review: “narrative summary”, which provides a descriptive overview and “repeated analysis”, which allows for additional analyses and comparisons [11]. The choice between these methods should be based on the purpose of the review, the topic area, and the characteristics of the included systematic reviews. The differences between these approaches are presented below and highlighted in Table 1.

**Table 1:** Main Differences between Narrative Summary and Repeat Analysis Approaches

Approach	Description	Purpose	Methodological Considerations
Narrative summary	Presenting data exactly as reported in included SRs, including narrative summaries and meta-analyzed data	Provide an overview of SR content and results, describe interventions, comparators, outcomes, and results	Extract and reformat effect estimates, 95% confidence intervals, and measures of heterogeneity from SR
Repeated analysis	Extracting relevant data from SRs and conducting different analyses than original reviews	Answer different clinical questions, apply consistent analyses, address differences in summary measures or models, analyze data not previously meta-analyzed	Adhere to standard meta-analytic principles, exercise caution to avoid inappropriate comparisons, understand reasons behind original review authors' analytic methods

Narrative summary

A narrative summary is a descriptive approach that synthesizes and presents the findings of the included systematic reviews or meta-analyses without further statistical analysis. This method is ideal when the focus is on providing an overview of the evidence, highlighting the range of findings, and identifying patterns, gaps, or variations across the studies. [21]

Repeated analysis

Repeated analysis involves conducting new statistical analyses on the data extracted from the included systematic reviews or meta-analyses. This approach allows for combining data to produce new insights and further exploration of the data, such as comparing outcomes across different reviews or conducting subgroup analyses. [22]

Finally, a clear and concise summary of the results is presented, utilizing tables, figures, or graphs to present the synthesized data and highlight key findings.

### Managing overlapping systematic reviews

Managing overlapping systematic reviews is crucial in an umbrella review [22], as they can share some or all of the same primary studies, leading to double counting and inflated findings [23]. Identifying overlaps is essential to avoid bias and accurately synthesize evidence.

To identify overlaps, researchers can examine the list of primary studies included in each systematic review and create a matrix where each row represents a primary study and each column represents a systematic review [24, 25], marking where studies overlap.

Several strategies can be employed to manage identified overlaps [11, 26]:

- ***Combining overlapping systematic reviews:*** If reviews are similar in the research question, population, interventions, and outcomes, they can be merged into a single review for the umbrella analysis.
- ***Selecting the most comprehensive or highest quality review:*** If merging is not feasible, choose the most comprehensive or highest quality review based on the number of included studies or methodological quality.
- ***Adjusting the data:*** In cases where it is essential to include all the overlapping systematic reviews, statistical methods can be used to adjust the data to account for the overlap. For instance, one might use a weighted average of the effect sizes from the overlapping systematic reviews.
- ***Narrative approach:*** If quantitative adjustments aren’t possible, qualitatively describe the overlaps and consider them in the findings' interpretation.

It’s essential to transparently report overlapping systematic reviews and the management approach to ensure the validity and robustness of the umbrella review's findings. Careful identification, strategic handling, and transparent reporting are key to providing a clear and unbiased evidence synthesis.

### Risk of bias assessment and grading the quality of evidence:

In conducting an umbrella review, it is an important consideration to evaluate the methodological quality and risk of bias of the included systematic reviews. This step ensures the credibility and reliability of the synthesized evidence. At least two reviewers should independently conduct quality assessments.

Researchers can utilize established tools such as AMSTAR, AMSTAR2, and ROBIS to achieve this.

1. **AMSTAR (A MeaSurement Tool to Assess systematic Reviews):** AMSTAR was developed in 2007 and is designed to appraise the methodological quality of systematic reviews focusing on randomized controlled trials (RCTs). It consists of 11 items that cover various aspects of the review process, including study selection, data extraction, and consideration of publication bias. AMSTAR has been proven to be a reliable and valid tool for evaluating the quality of systematic reviews [27].
2. **AMSTAR2:** An updated version of AMSTAR, AMSTAR2 expands its scope to assess the methodological quality of systematic reviews that include both randomized and non-randomized studies of healthcare interventions. It contains 16 domains in total, including 10 from the original tool. This tool includes items such as protocol registration, adequacy of literature search, risk of bias assessment, and appropriateness of meta-analytic methods. In addition, AMSTAR2 provides criteria for rating the overall confidence in the study, enabling a more comprehensive evaluation [28].
3. **ROBIS (Risk of Bias Assessment Tool for Systematic Reviews):** ROBIS was developed in 2016 to assess the risk of bias in systematic reviews. It addresses questions related to interventions, diagnosis, prognosis, and etiology studied in the reviews. ROBIS consists of three phases: assessing relevance, identifying concerns with the review process, and judging the overall risk of bias. This tool offers a valuable framework for evaluating potential biases in systematic reviews [29].

For analyzing the quality of evidence researchers can utilize established tools like **GRADE (Grading of recommendations, Assessment, Development, and Evaluations) system**, which offers a systematic approach for rating evidence in systematic reviews and the strengths of recommendations. [17, 18]. It is advisable to use GRADE alongside other tools for a comprehensive evaluation [2].

## Enhancing efficiency in umbrella reviews: an overview of specialized software tools

Systematic reviews and umbrella reviews are vital to evidence-based practice but are time-consuming and resource-intensive [30]. Specialized software tools can enhance efficiency in literature search, screening, data extraction, quality assessment, and data synthesis. These tools can significantly reduce the time spent on extensive searches for eligible studies, potentially saving up to 40% of the time required for manual screening [31].

Rayyan is a web-based tool designed to streamline initial systematic review stages, allowing for collaboration and reducing bias. It uses machine learning to predict study relevance, though it may not integrate seamlessly with other software, potentially requiring additional steps for data transfer and management [32]. **Covidence** is a comprehensive software used by healthcare and academic institutions for systematic reviews facilitating study selection, data extraction [33]. These tools can be effectively applied in umbrella reviews. However, some disadvantages have been reported, and the decision to choose one over the other could be case-specific and influenced by various factors.

At the data extraction stage, several models have been developed to facilitate the semi-automation of this process. While these models have been effective in extracting straightforward data such as PICO elements, not all are publicly accessible, and their utility in handling more complex data remains limited [34]. **ExaCT** could serve as an example of these systems.

Data synthesis in umbrella reviews can be performed through meta-analysis by pooling effect sizes from published systematic reviews or meta-analyses. In some cases, it may be necessary to re-analyze data from the original studies identified by previous SRs/MA, particularly if additional insight or new analyses are needed. The decision to use one approach over the other depends on the availability and quality of data, as well as the specific goals of the review.

## Discussion and conclusion

**Fig. 3:**
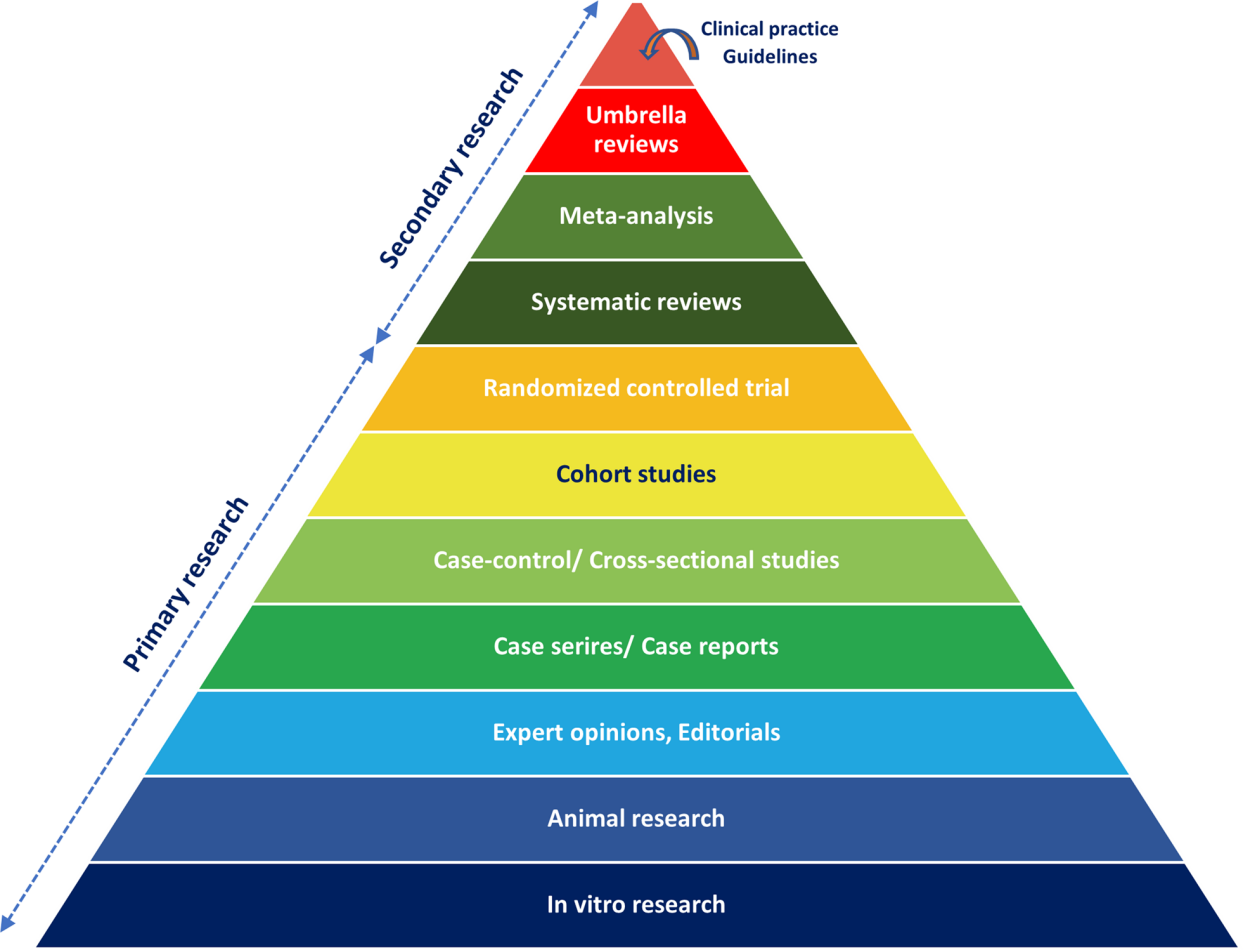
Hierarchy of scientific research design

Although systematic reviews are located at a high level in the hierarchy of scientific evidence (Fig. 3), as they provide reliable data synthesized from multiple studies, the exploding number of systematic reviews makes it difficult for readers to follow and update new knowledge. As a result, the “umbrella review” provides an excellent way to combine multiple systematic reviews. Umbrella reviews represent a higher level in the evidence hierarchy. They use two main analytical methods: narrative summaries, which descriptively map findings to identify consensus or gaps without further statistical analysis, and repeated analyses, which re-extract and re-analyze data to explore new questions or validate existing results. Since the umbrella review is a relatively new concept in the research field, we aimed to help young researchers, as well as anyone who wants to update their knowledge in research, by providing a step-by-step approach on how to conduct an umbrella review. If you are familiar with conducting systematic reviews, you may find many similarities to conducting umbrella reviews. Yet, the fundamental difference between systematic reviews and umbrella reviews is that only systematic reviews are included in an umbrella review.

One specific challenge of an umbrella review is the possible overlapping of primary studies in the included systematic reviews. This problem can cause biases and inflate the results. To identify the overlapping systematic reviews, we proposed a matrix approach. We can manage this problem by combining those studies, selecting the highest quality reviews, adjusting the data, or using a narrative approach. For quality assessment, there are three available tools: AMSTAR, AMSTAR2, and ROBIS. While the most common tool is AMSTAR, researchers should consider which tool to use based on their study design and preferences. 

We also reviewed software like Rayyan and Covidence for screening and ExaCT for data extraction. We anticipate that advancements in AI will lead to more automated applications, further reducing the time needed for umbrella and systematic reviews.

## Supplementary Information

Supplementary Material 1.

## Data Availability

No datasets were generated or analysed during the current study.
